# Immunological Targets in Generalized Myasthenia Gravis Treatment: Where Are We Going Now?

**DOI:** 10.3390/brainsci15090978

**Published:** 2025-09-11

**Authors:** Elena Rossini, Luca Leonardi, Stefania Morino, Giovanni Antonini, Laura Fionda

**Affiliations:** 1 NESMOS Department, Sapienza University of Rome, 00185 Rome, Italy; elena.rossini@uniroma1.it; 2 Neuromuscular and Rare Disease Center, Sant’Andrea Hospital, 00185 Rome, Italy; lleonardi@ospedalesantandrea.it (L.L.); smorino@ospedalesantandrea.it (S.M.); vanni.antonini@gmail.com (G.A.); 3 UniCamillus-Saint Camillus International University of Health Sciences, 00131 Rome, Italy

**Keywords:** generalized myasthenia gravis, immunopathology, autoantibodies, biological therapies

## Abstract

Background: Generalized myasthenia gravis (gMG) is a heterogeneous autoimmune disorder marked by antibody-mediated disruption of neuromuscular transmission. Despite advancements in immunosuppressive therapies and biologics, a subset of patients remains refractory, necessitating more targeted and personalized treatment strategies. Objective: This review aims to synthesize current knowledge of the immunopathological mechanisms across gMG subtypes and to explore emerging therapeutic targets tailored to these diverse disease phenotypes. Methods: A narrative review was conducted, integrating recent findings from clinical trials, immunogenetic studies, and preclinical research to describe subtype-specific immune mechanisms and corresponding therapeutic innovations. Results: gMG subtypes—characterized by autoantibody profiles (AChR, MuSK, LRP4, or seronegative), thymic histopathology, and age of onset—demonstrate distinct immunological pathways. Early-onset MG is associated with thymic hyperplasia and Th17-driven inflammation; thymoma-associated MG involves central tolerance breakdown; late-onset MG shows immune senescence and altered T-cell regulation. MuSK- and LRP4-positive MG exhibit unique cytokine and antibody signatures. Novel therapeutic strategies include B cell- and T cell-targeted therapies (e.g., anti-CD19, anti-CD38, JAK inhibitors), cytokine inhibitors (IL-6, IL-17, IL-23), FcRn antagonists, complement inhibitors, and gene- or cell-based therapies such as CAR-T and CAAR-T cells. Conclusion: The evolving landscape of gMG treatment reflects a shift toward immunopathology-based precision medicine. Better characterization of subtype-specific molecular signatures and immune dysfunctions is essential to guide clinical decision-making and improve outcomes for treatment-refractory patients.

## 1. Introduction

Myasthenia gravis (MG) is a chronic, disabling condition characterized by a fluctuating course, often resulting in difficulties in managing activities of daily living and leading to unpredictable hospitalizations. These challenges, combined with treatment-related adverse effects and tolerability issues, frequently exert a substantial negative impact on patients’ quality of life [1,2].

The primary therapeutic goal in the management of MG is to achieve minimal manifestation status or clinical/pharmacological remission, as defined by the Myasthenia Gravis Foundation of America Post-Intervention Status (MGFA-PIS). This should be accomplished while minimizing the daily corticosteroid dose and maintaining an acceptable side-effect profile from adjunctive therapies [3].

To evaluate progress toward these goals, clinical outcomes are typically assessed using standardized tools such as the Myasthenia Gravis Activities of Daily Living (MG-ADL) [4] and the Quantitative Myasthenia Gravis (QMG) [5] scores. The MG-ADL is practical and sensitive to changes in daily functioning but is inherently subjective and more focused on bulbar symptoms, limiting its ability to capture the full heterogeneity of the disease. In contrast, the QMG offers a more objective assessment of muscle strength but is time-consuming and susceptible to inter-rater variability. Additional instruments, such as the Myasthenia Gravis Composite (MGC) [6] and the revised MG Quality of Life 15 (MG-QoL15r) [7], complement these core measures by incorporating both physician and patient perspectives or focusing specifically on health-related quality of life. Nevertheless, each scale has intrinsic limitations and reflects only a portion of the overall disease burden, underscoring the need for integrated assessment strategies and the development of novel biomarkers to more accurately capture treatment response.

Although conventional therapies for generalized MG (gMG)—including acetylcholinesterase inhibitors, corticosteroids, and commonly used immunosuppressants such as azathioprine, mycophenolate mofetil, and cyclosporine—are effective in controlling symptoms in most patients, a subset continues to experience significant disease burden due to incomplete response, treatment-related adverse effects, and disease refractoriness. This unmet clinical need has stimulated the development of novel therapeutic strategies [8].

Several pharmacological agents have been investigated—and continue to be evaluated—in clinical trials for the treatment of gMG, with some biologics having already received regulatory approval. Among them, biologics such as the complement inhibitors eculizumab, ravulizumab, and zilucoplan and the FcRn blockers efgartigimod, rozanolixizumab, and nipocalimab have already received regulatory approval in recent years. These agents have demonstrated clinical efficacy in approximately 60–80% of patients; however, a subset of individuals remains refractory even to these advanced therapies [9,10]. Furthermore, limited data are available on the feasibility of tapering or discontinuing concomitant immunosuppressive treatments, as well as on the clinical and immunological characteristics of this ultra-refractory subgroup [11]. These uncertainties further complicate treatment decisions, particularly in elderly patients, among whom the prevalence and recognition of MG are increasing, and comorbidities are also very common [12].

Importantly, age at disease onset correlates with distinct MG subtypes, which differ in terms of disease severity, sex distribution, and thymic histopathology. Although these subtypes may present with similar ocular and/or generalized symptoms, their underlying immunopathological mechanisms are markedly heterogeneous [13,14]. This heterogeneity is reflected in differences in autoantibody profiles, response to therapies, and HLA-associated susceptibility alleles. Consequently, MG is now recognized as a biologically diverse disorder [15].

Accurate identification of these clinical subtypes is critical, as therapeutic strategies are often tailored accordingly. For instance, the indication for thymectomy and the selection of certain biologic therapies depend on the patient’s specific MG subtype [9,16]. Moreover, it is plausible that other, as yet unidentified factors in the immunological cascade specific to each subtype may serve as novel therapeutic targets. Some of these mechanisms may have been explored only in preclinical models, underscoring the need for further translational research to expand the repertoire of effective immunotherapies.

A detailed examination of the various factors contributing to the pathophysiological background of gMG may offer critical insights into the rationale for the development of targeted therapies.

In this review, following PRISMA guidelines, we aim to provide a focused overview of the current understanding of the pathophysiological mechanisms underlying the different gMG subtypes and explore how these distinctions may influence therapeutic decision-making. Emphasis will be placed on emerging experimental targets and novel therapeutic strategies under investigation, which may pave the way for more personalized and effective treatment approaches.

## 2. Classification of gMG

MG is classified according to autoantibody status, most commonly into anti-acetylcholine receptor (AChR) (85%), anti-muscle-specific kinase (MuSK) (6%), and anti-low-density lipoprotein receptor-related protein 4 (LRP4) (2%) subtypes, as well as seronegative MG [15]. Although rare, LRP4 antibodies can also be detected in AChR-positive and MuSK-positive cases. In one study, approximately 7.5% of AChR-MG and 15% of MuSK-MG patients were found to be double positive for anti-LRP4 [17]. Seronegative MG is diagnosed when clinical and electrophysiological findings are consistent with MG, but repeated testing—including cell-based assays (CBAs)—fails to detect known pathogenic autoantibodies [18].

Among anti-AChR-positive patients, further sub-classifications are recognized: early-onset MG (EOMG), late-onset MG (LOMG), and thymoma-associated MG (TAMG), each representing distinct clinical and immunopathological entities [15].

Autoantibodies play a central role in the pathogenesis of MG due to their well-established direct pathogenic effects at the neuromuscular junction (NMJ) [15]. However, the upstream mechanisms driving the production of these autoantibodies—and the broader breakdown of immune tolerance—remain poorly defined, particularly when analyzed within the context of MG subtypes. Understanding these mechanisms is critical for advancing therapeutic approaches.

Current approved biologic therapies—and those in late-stage development—primarily act by inhibiting downstream immunological pathways. These include complement cascade inhibition (e.g., eculizumab, ravulizumab) and neonatal Fc receptor (FcRn) blockade, which accelerates IgG degradation. While these agents produced robust results in clinical trials and demonstrated rapid onset of their clinical efficacy, their capacity to induce long-term immunomodulatory effects remains uncertain, and they are currently indicated only as adjunctive treatments. Updated clinical guidelines support their use in selected patient populations, especially in cases of refractory disease to conventional immunosuppressive treatments, while also acknowledging their high cost and limited long-term data [19,20].

Recent treatment recommendations emphasize the role of biologics as add-on therapies rather than as monotherapies. Most patients are advised to continue corticosteroids and/or steroid-sparing immunosuppressants, with the goal of tapering corticosteroids to the lowest effective dose without complete withdrawal [21,22].

Notably, in phase III randomized controlled trials (RCTs) evaluating both complement inhibitors and FcRn antagonists, up to 90% of participants remained on background corticosteroids and/or non-steroidal immunosuppressive therapies (NSISTs) throughout the study period. These findings have been echoed in real-world studies, which further suggest heterogeneity in treatment responses across MG subtypes [9,10,23,24,25]. For example, age of onset and the presence of thymoma appear to influence therapeutic outcomes with newer agents [26,27].

This variability highlights the importance of understanding the distinct immunopathogenic mechanisms underlying each MG subtype. Treatments effective in one subgroup may lack a mechanistic rationale—or clinical benefit—in another. As such, precision in diagnosis and disease classification is essential for individualized therapy.

Before addressing emerging and under-explored therapeutic targets in gMG, we will first review the immunopathogenic features known to characterize each major subtype, with a focus on how these mechanisms may inform treatment selection and development.

### 2.1. EOMG

Patients in the EOMG category have the onset of disease before the age of 50 and are often women.

The female predominance observed in many autoimmune diseases may be partly explained by estrogens, which reduce the expression of the autoimmune regulator (AIRE) gene in the thymus. AIRE encodes a transcription factor in medullary thymic epithelial cells (mTECs) that promotes the expression of self-antigens, including CHRNA1, which encodes the acetylcholine receptor α-subunit. In the thymus, these self-antigens are presented to developing T cells so that autoreactive cells can be eliminated or diverted into regulatory T cells. When this process is disrupted, AChR-specific T cells may escape central tolerance and drive autoimmunity [28]. Recent evidence also suggests that unbalanced X-linked transcription caused by skewed X-chromosome inactivation (XCI) may impair immune tolerance. During normal development, random XCI in dendritic cells allows balanced expression of parental X-linked self-antigens, which is crucial for the negative selection of autoreactive T cells in the thymus. Skewed XCI patterns, in line with the loss of mosaicism hypothesis, may compromise this process and promote the release of autoreactive immune cells into the circulation [29].

Patients with EOMG usually have conspicuous morphological changes in the thymus, characterized by follicular hyperplasia, especially in the medullary region, where B cells and antibody-secreting cells (ASCs) organize into structures called germinal centers (GCs). These structures are observed in approximately 70% of EOMG patients and play a central role in EOMG pathogenesis [14,30,31]. By contrast, thymic GCs are typically absent in late-onset MG (LOMG) without thymoma, which is consistent with the age-related thymic involution commonly observed after middle age [32].

It is unclear whether thymic dysfunction is indeed the primary causal event of the disease. It has been hypothesized that, following a triggering event (e.g., infectious agents leading to molecular mimicry) [33] on a genetically favorable background [34], an overproduction of cytokines and chemokines driven by thymic epithelial cells (TECs) and T lymphocytes could start the immunological cascade. TECs, through their expression of tissue-specific antigens, induce thymocyte maturation/differentiation processes and are involved in the development of self-tolerance education, as well as modulating CD4^+^CD25^+^ regulatory T cell (Treg) formation. Dysfunction of Tregs could also contribute to the development of a favorable background for the escape of autoreactive T cells and the maintenance of a pro-inflammatory environment.

Viruses and Toll-like receptors (TLRs), whose role in the innate immune system is crucial in recognizing and responding to invading pathogens, have been implicated in triggering the immunological cascade of MG within the thymus [35,36]. TLRs (in particular, TLR3 and TLR7), after being activated by pathogens, induce the overproduction of pro-inflammatory cytokines, such as interferon β (IFN-β), and various chemokines that promote AChR expression by TECs and their uptake by antigen-presenting cells (APCs), leading to the autoimmunization against AChR [37,38].

TECs are also involved in the overproduction of cytokines, especially interleukin-1 and -6 (IL-1, IL-6) and transforming-growth-factor-β (TGF- β), that alter thymocyte differentiation and participate in the development of CD4^+^ T helper cells (Th) responses toward AChR [31,39]. Among these, Th17 cells produce other pro-inflammatory cytokines, including interleukin-17 (IL-17) and interleukin-22 (IL-22). There is evidence that the number of Th17 cells and levels of IL-17 are higher in AChR-MG patients compared to healthy controls and that they correlate with the severity of the disease [34]. Th17 cells induce B cells to transform into APCs and memory B cells, whose amount is effectively increased in the hyperplastic thymus.

Other CD4^+^ Th cell subclasses are also involved in the inflammatory process that imbalances the reduction of Tregs and B-cell recruitment, proliferation, and differentiation. Th1 cells produce interleukin-2 (IL-2), interferon γ (IFN-γ), and tumor necrosis factor- α (TNF-α), which enhance the proliferation of other CD4^+^ subclasses through the exposure of epitopes on AChRs. Functional studies suggest that TNF-α plays a role in maintaining the chronic inflammation observed in the MG thymus [31,40]. T follicular helper (Tfh) cells secrete interleukin-21 (IL-21), promoting self-differentiation and enhancing the expression of C-X-C Motif Chemokine Receptor 5 (CXCR5) and B-cell lymphoma 6 (BCL-6), which stimulates the migration of B cells to GCs and therefore the differentiation and proliferation of B cells through CD40-CD40L interaction [31]. These cytokines also increase the expression of MHC class I and II and chemokines, such as C-X-C motif Chemokine ligand 13 (CXCL13) (the receptor of CXCR5), C-C motif Chemokine ligand 21 (CCL21), and stromal cell-derived factor 1 (SDF-1), that actively recruit peripheral B cells and Th17 cells [41]. CXCL13 is selectively chemotactic for B cells and is produced by TECs, Tfh cells, and also by B cells; CCL21 participates in prothymocyte recruitment in the thymus, and its overexpression is primarily found in the lymphatic vessels, together with SDF-1 (involved in B- and T-cell homing and GC organization) [42,43].

Once recruited from the peripheral circulation, transitional or mature B cells become activated through interactions with CD4^+^ T cells, leading to somatic hypermutation and clonal selection [43,44].

In particular, transitional B cells can mature into a follicular type I (FO-I) or type II (FO-II) B cell, induced either by B cell-activating factor (BAFF) and/or Bruton’s tyrosine kinase (BTK), and can then migrate to secondary lymphoid organs, where they generate GCs. Transitional B cells mature into Fo-I B cells if they recognize a self-antigen with high affinity, whereas self-reactive B cells with a lower affinity can either mature into marginal zone (MZ) B cells or into recirculating mature Fo-II B cells that might serve as a reservoir for MZ B cells when they are rapidly depleted by blood-borne pathogens [45].

Different factors are implicated in the maturation and differentiation of B cells within the hyperplastic thymus: increased levels of BAFF and a proliferation-inducing ligand (APRIL) create a microenvironment that supports the survival and proliferation of autoreactive B cells within ectopic GCs. BAFF is a member of the TNF family secreted by macrophages and dendritic cells that is available at high concentrations in lymphoid follicles, where it functions as a survival factor for newly generated transitional B cells and mature follicular B cells. BAFF is also required for MZ B-cell development, a requirement that seems to be separate from its role in promoting the survival of B cells in the follicle. APRIL shares with BAFF the function of supporting B-cells’ survival and proliferation, as well as two receptors: BCMA, expressed by B cells and plasma cells; and transmembrane activator and cyclophilin-induced ligand (TACI), present on T cells. BAFF, but not APRIL, signals via the BAFF-receptor (BAFF-R) present on B and T cells, a pathway that promotes survival in lymphocytes. APRIL, but not BAFF, binds CD138, expressed by plasma cells [46].

During the maturation process, FO-I (and partially FO-II) B cells begin to produce high-affinity, class-switched antibodies and differentiate into long-lived plasmablasts, which are responsible for T-cell-mediated response to pathogens and serve as the main source of persistent high-affinity autoantibody production. MZ B cells differentiate into short-lived plasma cells that produce low-affinity antibodies (mostly IgM but also IgG4), providing a quick, albeit temporary, defense (T-cell dependent in follicles or T-cell independent in bone marrow). Short-lived plasmablasts are often described as CD20^−^ CD19^med/+^ IgD^−^ CD27^hi^ CD38^hi^ (of those, some but not all are also CD138^+^), while long-lived plasma cells are described as CD20^−^ CD19^−/+^ CD38^hi^ CD138^+^ [45,47].

Long-lived ASCs can migrate to the bone marrow and secrete high-affinity, class-switched antibodies (as needed, with memory B cells). In summary, long-lived plasma cells are essential for sustaining serum antibody concentrations over time, while memory B cells mediate rapid recall responses upon re-exposure to the same antigen [48].

Short-lived plasma cells develop in extrafollicular regions of secondary lymphoid organs [49], as during infections or inflammation, B-cell numbers increase in peripheral blood if induced by tissue-resident memory T cells, which can activate resident B cells and initiate an early plasmablast response [50].

Of note, in the MG hyperplastic thymus, activated B cells themselves spontaneously induce the synthesis of anti-AChR antibodies, as demonstrated in vitro [30] and in mouse models, where AChR-MG thymic tissue can determine AChR-specific antibody production and can cause muscle weakness [51].

In summary, EOMG is characterized by thymic follicular hyperplasia with GC formation, where aberrant interactions between CD4^+^ T cells and B cells sustain autoantibody production. The immunopathology reflects a complex cytokine network: IL-6 promotes B-cell differentiation and the expansion of Th17 cells; Th17-derived IL-17 and IL-22 drive pro-inflammatory responses and correlate with disease severity; Th1-derived IFN-γ and TNF-α amplify antigen presentation and maintain chronic thymic inflammation; and Tfh cells secrete IL-21, which supports B-cell proliferation and differentiation through CD40–CD40L interactions. In parallel, BAFF and APRIL provide survival signals for autoreactive B cells, while chemokines such as CXCL13, CCL21, and SDF-1 recruit lymphocytes and organize ectopic GCs. Collectively, these mechanisms converge to promote the maturation of autoreactive plasma cells and the persistent production of pathogenic anti-AChR antibodies in EOMG.

### 2.2. TAMG

TAMG typically occurs around 50 years of age with generalized features, without a sex prevalence [52]. Even if there is no major gender bias and no strong HLA association, loss of heterozygosity at the HLA locus at 6p21 is described in some cases of TAMG and can occur in all histological thymoma subtypes [53]. Polymorphisms of non-HLA genes can also affect T-cell receptor signaling (e.g., CTLA4, PTPN22) and seem to correlate with TAMG, impairing thymus negative selection for self-tolerance [54].

Thymoma is present in 10–20% of MG patients, predominantly AB or B subtype, while up to 50% of thymoma patients develop MG, almost always associated with anti-AChR autoantibodies. The frequency of other autoimmune diseases, as well as immunodeficiency, is higher than in other MG subtypes and also than in other cancer-associated paraneoplastic diseases [55]. In TAMG, the cortical regions show increased numbers of neoplastic epithelial cells that are intermingled with numerous T cells. By contrast, the medullary regions show rudimentary development, only rare B cells and lymphoid follicles, some Hassall corpuscles, and the near absence of AIRE-positive epithelial cells, Treg cells, and myoid cells [56].

The immunopathology of TAMG is characterized by a breakdown of central tolerance within the thymic microenvironment, leading to the generation of autoreactive T and B cells that target NMJ antigens, ectopically expressed by aberrant medullary TECs in thymoma. These antigens include AChR subunits, titin, and the ryanodine receptor, which are not normally presented in this context. This aberrant expression facilitates the presentation of self-antigens to developing thymocytes, promoting the escape and peripheral export of autoreactive T cells, and is located in the cortico-medullary region [57,58,59].

Defective expression of the AIRE gene and MHC class II molecules in thymomas further impairs negative selection, compounding the loss of self-tolerance [52]. Near-complete loss of AIRE expression in TAMG, leading to more profound central tolerance failure, identifies a distinct immunopathogenic mechanism of this subgroup, also associated with a worse prognosis [53]. Moreover, Th17 cells were increased and Tregs decreased in both the thymoma and peripheral blood of TAMG patients [60].

Cortical thymomas resemble the thymic cortex in morphology and can produce polyclonal CD4^+^ and CD8^+^ thymocytes from bone marrow progenitors, which are then released into the bloodstream. It is important to note that thymopoietically incompetent thymic carcinomas are not associated with MG. Moreover, once the disease is initiated, it remains self-sustaining even after complete removal of the thymoma [61].

The presence of intratumor naive CD4^+^ T cells is a distinctive feature of TAMG, as naive CD4^+^ [62] T cells are significantly reduced in thymomas without MG. The medullary region may also show distinctive features in TAMG compared to thymoma without MG, characterized by a specific chemokine pattern and immune cell composition, including migratory dendritic cells, Treg cells, high endothelial venules, and GCs, similar to what is observed in the thymic hyperplasia medulla, even if less pronounced [58]. The presence of GCs and high endothelial venules is associated not only with MG but also with younger age at MG symptom onset and higher levels of anti-AChR autoantibodies [63].

TAMG also features an imbalance in CD4^+^ T-cell subsets, with increased Th17 cells and decreased Tregs, driven by elevated levels of IL-6 and IL-21, which further sustain inflammation and autoimmunity [60,64,65]. Collectively, these mechanisms result in the production of pathogenic anti-AChR antibodies and other neuromuscular autoantibodies, leading to the clinical manifestations of MG in patients with thymoma [57,66].

Thymomas from MG patients express high levels of type I IFNs [31]. IFN-γ and IL-10 were upregulated in both EOMG and TAMG patients, while IL-1β and sCD40L were only detected at significantly higher levels in the sera of TAMG patients, suggesting a robust link between CD40 pathway activation and disease burden in this subgroup [63]. In contrast, in non-thymomatous MG, peripheral blood CD40 expression on immune cells does not show a consistent or strong correlation with disease severity, and the immunopathogenic mechanisms are less dependent on thymic germinal center activity and CD40 signaling. Thus, the relationship between CD40 expression and MG severity is most pronounced in TAMG, where local thymic immune dysregulation and CD40 pathway activation play a central role in driving severe, antibody-mediated disease [67,68].

In thymomas, APRIL-positive cells, as well as BAFF-R-positive cells, seem to be rare, while BAFF expression is diffuse. Additionally, B-cell maturation antigen (BCMA)-positive cells are scattered and show a distinct cytoplasmic staining, while they are more abundant in hyperplastic thymi [46].

In summary, TAMG is driven by central tolerance failure within the tumor microenvironment, where defective expression of the AIRE and MHC class II molecules allows the escape of autoreactive T cells. Imbalances in T-helper cell subsets—particularly increased Th17 cells and decreased Treg cells—create a pro-inflammatory milieu. Elevated levels of IL-6, IL-21, IFN-γ, and IL-1β enhance antigen presentation and sustain T-cell activation. Tfh cells, through IL-21 and CD40–CD40L signaling, promote B-cell proliferation and autoantibody production. Although thymomas show GC formation compared with EOMG, increased sCD40L in sera suggests ongoing T–B collaboration. The combined breakdown of central and peripheral tolerance in TAMG results in the generation of pathogenic anti-AChR antibodies and other neuromuscular autoantibodies.

### 2.3. LOMG

LOMG, typically defined as onset after age 50, is associated with thymic atrophy prevalence and mostly occurs in males. The immunopathogenesis determines a predominance of anti-AChR antibodies (also compared with EOMG), with a higher prevalence of titin and ryanodine receptor antibodies, which are markers of more severe disease in this subgroup. The HLA associations also differ from EOMG, and it is linked to HLA-DR2, HLA-B7, and HLA-DRB1*15:01 haplotypes, suggesting a distinct genetic predisposition [69,70].

The thymus shows normal-for-age atrophy but is characterized by a paucity of muscle-like myoid cells, which may contribute to the generation of muscle-directed autoantibodies, and autoimmune regulator-positive TECs, as can be found in thymomas [56].

Recent genomic studies have identified that LOMG is specifically associated with genetic variants affecting T-cell regulators, particularly CD28 and CTLA4. These variants result in reduced CTLA4 expression in CD4^+^ T cells, which impairs T-cell regulation and promotes autoimmunity. This differs from EOMG, where genes involved in innate immunity as well as TLR signaling are more prominent. The immunological environment in LOMG is further shaped by age-related immune senescence, leading to altered T-cell function and increased autoreactivity [71]. Supported by dysfunctional T-cell regulation, B cells are induced to differentiate into plasma cells that secrete pathogenic autoantibodies [72].

Even within an atrophic thymus containing a nest of cells, APRIL-positive macrophages, BAFF-R-positive B cells, and CD138-positive plasma cells have been found. Some non-specific BAFF staining of atrophic thymus was also observed [46]. Of note, APRIL levels are particularly increased in patients with non-thymomatous LOMG when compared with TAMG and EOMG [73].

In summary, LOMG is characterized by thymic atrophy and age-related immune senescence. Genetic variants affecting T-cell regulation reduce inhibitory signals and promote autoreactivity. Increased IL-6 and TGF-β drive Th17 polarization, while impaired Treg function weakens tolerance. BAFF and APRIL support plasma cell survival and sustained autoantibody production, contributing to the more severe clinical phenotype and frequent additional autoantibodies observed in this subgroup.

### 2.4. MuSK-MG

MuSK-MG accounts for 1 to 10% of cases, with female predominance and onset before the fifth decade [74]. This disorder is more common in the Mediterranean area of Europe than in northern Europe, while it is also more common in the northern regions of East Asia than in the southern ones [75]. This variation is thought to be dependent on genetic predisposition rather than environmental factors. Patients with MuSK antibodies show a distinct clinical picture with more severe weakness, sometimes muscle atrophy, and marked facial and bulbar symptoms. Limb and ocular involvement are less common, and fluctuations in muscle strength are less pronounced than in anti-AChR-MG [69].

Unfortunately, data on lymphocytes and cytokines in anti-MuSK antibody-positive MG are scarce, presumably because of the relative rarity of this type of MG. T cells play a critical role in the immunopathology of MuSK-MG, particularly in supporting B-cell dysregulation and the production of pathogenic autoantibodies. In MuSK-MG, there is evidence of aberrant Th-cell activity, especially involving Th1, Th17, and Tfh cells. In vitro studies demonstrate that peripheral blood mononuclear cells from MuSK-MG patients secrete increased levels of Th1 (IFN-γ), Th17 (IL-17A), and Tfh (IL-21) cytokines upon stimulation, indicating a heightened pro-inflammatory and B cell-helping environment compared to healthy controls and AChR-MG patients [76,77]. Tfh cells are particularly important as they provide essential signals (e.g., IL-21, CD40L) for B-cell activation, class switching, and differentiation into short-lived plasmablasts, which are the main producers of MuSK autoantibodies [78].

Although CD40L expression on CD4^+^ T cells is reduced in MuSK-MG, the functional capacity of Tfh cells to support B-cell responses appears preserved or even enhanced, as reflected by increased IL-21 secretion [76,77]. This T cell–B cell interaction is central to the generation of the IgG4-dominant autoantibody response characteristic of MuSK-MG [78,79]. Additionally, there is evidence of broad immune dysregulation, with abnormalities in both naive and memory B-cell repertoires, likely driven by defective T-cell tolerance and regulatory T-cell dysfunction. These T-cell abnormalities facilitate the escape and persistence of autoreactive B cells, promoting ongoing autoantibody production and disease activity in MuSK-MG [78]. Upon activation, these autoreactive B cells differentiate predominantly into short-lived plasmablasts rather than long-lived plasma cells, which is distinct from the immunopathology seen in AChR-MG. These plasmablasts are the primary source of pathogenic MuSK autoantibodies, mainly of the IgG4 subclass. Specifically, anti-MuSK antibodies are predominantly IgG4 and disrupt the MuSK–LRP4 interaction, thereby impairing AChR clustering and signal transduction [79]. IgG4 antibodies are functionally monovalent due to Fab-arm exchange, and they exert pathogenicity by directly blocking the interaction between MuSK and LRP4, thereby inhibiting MuSK signaling at the neuromuscular junction [10,78].

Higher BAFF levels and lower percentages of B10 cells have also been reported in patients with anti-MuSK antibody-positive MG [80].

In summary, MuSK-MG is characterized by a distinct immunopathology driven by different subtypes of Th cells that provide crucial signals to B cells, determining class switching and the generation of short-lived plasmablasts that produce a specific class of pathogenic antibodies. Increased IL-17 and IFN-γ further contribute to a pro-inflammatory environment, while reduced regulatory B cells facilitate the persistence of autoreactive clones. These mechanisms explain the unique clinical phenotype and the predominance of IgG4 antibodies in this subtype, which block MuSK–LRP4 signaling at the neuromuscular junction.

### 2.5. LRP4-MG

The most common patients affected by LRP4-MG are young individuals, with a female predominance, with a mean age of onset in the early 30s for females and early 40s for males in European and Mediterranean populations, while over half of LRP4-MG cases occur in pediatric patients in Chinese cohorts [17,81]. The disease is generally milder, often presenting with ocular or mild generalized symptoms at onset, and is less commonly associated with thymic pathology or thymoma compared to AChR-MG or MuSK-MG [82,83,84].

Although direct studies on LRP4-MG pathophysiology are limited, the absence of thymic germinal center hyperplasia in most LRP4-MG patients suggests that peripheral lymphoid tissues may be the primary site of B cell–T cell interaction and autoimmunity [85]. The predominance of IgG1 subclass antibodies in LRP4-MG suggests a T cell-dependent, germinal center-driven humoral response [86]. Regulatory T-cell dysfunction, as described in other MG subtypes, may also contribute to loss of tolerance and sustained autoantibody production, while B cells are responsible for generating high-affinity, class-switched autoantibodies that disrupt the agrin-LRP4-MuSK signaling pathway at the neuromuscular junction.

In summary, LRP4-MG is usually associated with limited thymic pathology, suggesting that peripheral immunity sustains disease activity. Tfh-derived IL-21 supports B-cell maturation, while IL-6 and CXCL13 promote recruitment and follicle-like organization. These interactions lead to the production of IgG1/IgG2 antibodies against LRP4, highlighting the importance of peripheral T–B collaboration in this subtype.

### 2.6. Seronegative MG

Seronegative MG (SNMG) most commonly presents in younger patients, with a slight female predominance, and is more likely to manifest with mild to moderate generalized or ocular symptoms. Compared to antibody-positive subtypes, SNMG patients are typically younger at onset [87,88].

SNMG is less likely to be associated with thymic pathology or thymoma, and clinical features are generally mild. Ocular symptoms are common, and the disease course is often less severe than in MuSK-MG, but SNMG patients may have a higher disease burden due to diagnostic delays and suboptimal treatment response [18,89].

In seronegative MG, the immunopathology is less well defined. Recent studies show B-cell dysregulation with increased memory B cells, reduced soluble CD22, and fewer Treg cells, correlating with disease severity. However, the absence of detectable pathogenic autoantibodies suggests either undetected antibodies (due to assay limitations) or non-antibody-mediated mechanisms. T-cell dysregulation is present, with increased Th17 and Tfh activity, but the patterns are less distinct than in antibody-positive subtypes [90].

In summary, seronegative MG is characterized by subtle immune dysregulation, with increased Th17 and Tfh activity and reduced regulatory T- and B-cell functions. Cytokines such as IL-21 drive persistent immune activation, even in the absence of detectable autoantibodies. This imbalance suggests that cellular immunity and defective regulatory networks could play a central role in disease maintenance in this subgroup.

### 2.7. Similarities and Differences Among gMG Subtypes

At the core of the immunopathogenesis of MG lies a complex interplay between pathogenic autoantibodies and downstream immune effector mechanisms. The final common pathway in the development of MG symptoms involves the action of specific autoantibodies and, in certain subtypes, the activation of the complement system.

#### 2.7.1. Immunoglobulin Structure

ASCs produce large quantities of immunoglobulins (Igs), heterodimeric proteins composed of two heavy (H) chains and two light (L) chains. Igs are classified into five isotypes based on the structure and function of their heavy chains. In MG, all pathogenic autoantibodies identified to date belong to the IgG class. IgG antibodies are further subdivided into four subclasses—IgG1, IgG2, IgG3, and IgG4—based on structural, antigenic, and functional differences in the constant region of the heavy chain. These subclasses differ significantly in their Fc-mediated effector functions. All IgG subclasses can bind Fc gamma receptors (FcγRs) expressed on immune cells such as macrophages and natural killer (NK) cells, facilitating antibody-dependent cell cytotoxicity (ADCC) and antibody-dependent cellular phagocytosis (ADCP). However, the affinity for FcγRs varies: IgG1 and IgG3 exhibit high affinity for all FcγR types (I, II, and III), whereas IgG2 and IgG4 primarily bind FcγRII and III with markedly lower affinity [91]. The subclasses also differ in their ability to activate complement. IgG3 is the most potent at initiating complement-dependent cytotoxicity (CDC), followed by IgG1 and IgG2, while IgG4 lacks the ability to fix complement entirely. Consequently, the predominant IgG subclass in the circulating pool of autoantibodies shapes the pathophysiological mechanisms of the autoimmune response and has important therapeutic implications.

Of note, antigen recognition and neutralization are mediated by the Fab fragment of the IgG, and this function is largely conserved across subclasses. However, IgG4 is unique in that it can undergo Fab-arm exchange, resulting in bispecific and functionally monovalent antibodies. This structural feature makes IgG4 incapable of crosslinking antigens and triggering endocytosis or clustering.

While these antibody functions play a critical protective role in host defense under physiological conditions, in antibody-mediated autoimmune diseases, the same effector mechanisms contribute to tissue injury and functional disruption of target antigens, leading to the clinical manifestations and histopathological features of disease.

#### 2.7.2. Antibody Classes

MG is a prototypical antibody-mediated disease of the NMJ. Anti-AChR antibodies are primarily of the IgG1 and IgG3 subclasses, while antibodies against MuSK are predominantly of the IgG4 subclass. LRP4 autoantibodies are typically of the IgG1 or IgG2 subclass.

In AChR antibody-positive MG, which represents the most prevalent subtype, IgG1 and IgG3 subclass antibodies contribute to disease via multiple mechanisms: they block the interaction between acetylcholine and its receptor, promote internalization and degradation of AChRs through antibody cross-linking, and activate the classical complement pathway, resulting in structural damage to the NMJ [92].

In contrast, anti-MuSK antibody-positive MG involves predominantly IgG4 antibodies, which do not activate complement. MuSK, a postsynaptic receptor tyrosine kinase essential for the clustering and stabilization of AChRs at the NMJ, is normally activated by neural agrin through its co-receptor LRP4. This activation initiates a phosphorylation cascade critical for NMJ integrity [93].

In MuSK-MG, IgG4 autoantibodies disrupt the interaction between MuSK and LRP4, thereby blocking MuSK activation and impairing downstream signaling. This leads to the dispersal of AChR clusters, impaired synaptic transmission, and subsequent muscle weakness. Thus, their pathogenicity stems from direct functional blockade rather than immune-mediated destruction.

#### 2.7.3. Complement Cascade Activation

Focusing specifically on the complement cascade, the IgG1 and IgG3 subclasses initiate the classical pathway by binding C1q, a recognition protein that interacts with the Fc (CH2) domains of IgG. This binding activates the associated C1r and C1s subunits, converting them into active serine proteases. Notably, C1q also has a role in regulating T-cell and polymorphonucleate leucocyte https://www.sciencedirect.com/topics/immunology-and-microbiology/neutrophil-granulocyte (accessed on 7 September 2025) activation via specific C1q receptors. Activated C1s cleaves both C4 and C2, leading to the formation of the classical C3 convertase (C4b2a). This enzyme determines the cleavage of C3 into C3a and C3b, the latter of which can bind to C4b2a to form the C5 convertase (C4b2a3b).

C3b also participates in the alternative pathway. The binding of C3b to Factor B, which is then cleaved by Factor D into Bb, generates the alternative C3 convertase (C3bBb), which also plays a role in amplifying the cascade.

Factor B circulates as an inactive proenzyme and only becomes activated after cleavage by the protein Factor D, which can cleave Factor B only when it is bound to the active forms of C3. Factor D, a serine protease, circulates in the plasma as a mature but resting form and is produced mainly in adipocytes. The function of factor D is to cleave its unique substrate, Factor B, in a Mg^++^-dependent complex with C3b, to generate the alternative pathway C3 convertase. Though the classical pathway initiates the response in MG, the alternative pathway serves as an amplification loop, enhancing complement activation regardless of the initiating trigger. Therefore, Factor D is a key and rate-limiting component in the alternative pathway, but its participation in the amplification loop contributes significantly to responses elicited by the complement classical and lectin pathways.

The C5 convertase cleaves C5 into C5a and C5b. C5b then sequentially recruits complement proteins C6, C7, C8, and multiple copies of C9 to assemble the membrane attack complex (MAC, or C5b-9). This MAC inserts into the postsynaptic membrane, forming transmembrane pores that lead to ionic imbalance, cellular damage, and, ultimately, lysis of the postsynaptic membrane [94].

Given that antibody-mediated complement response is driven exclusively by the classical pathway, targeting the classical pathway components could have some advantages. Inhibiting the classical pathway can block complement-mediated damage while preserving MAC formation through the lectin and alternative pathways, potentially maintaining host defense. Additionally, classical pathway inhibition may exert immunomodulatory effects, particularly through its regulatory interactions with T cells and polymorphonuclear leukocytes via C1q and its receptors.

## 3. Biological Targets

In this section, we will focus on emerging experimental targets and novel therapeutic strategies under investigation. Table 1 provides an overview of the biological targets in MG and the associated therapeutic agents, including their mechanisms of action, key clinical findings, and trial status.

### 3.1. T Cell- and Cytokine-Targeted Therapies

**Figure 1:**
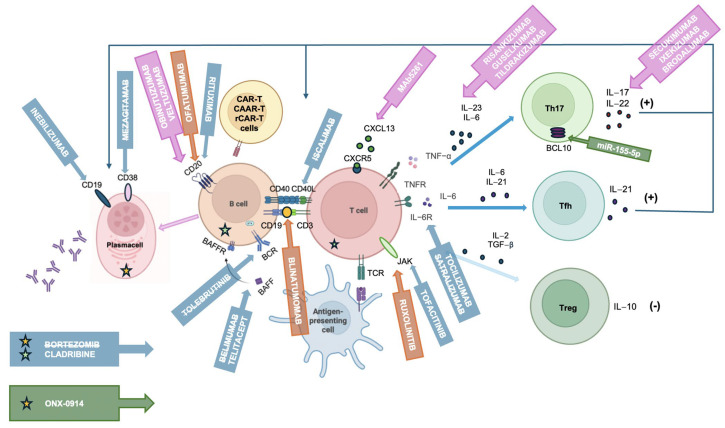
B and T cell immunologically targeted therapies. This figure illustrates the key targets and corresponding therapies aimed at B and T cells. The figure is intentionally schematic to facilitate understanding and highlight the main therapeutic strategies. Experimental drugs with evidence from preclinical models are shown with green arrows; agents already used in other diseases with targets potentially relevant for MG are highlighted in pink; drugs evaluated in RCTs are highlighted in blue; and those reported in case reports or case series are highlighted in orange. The white line crossing the drug name indicates agents that did not show favorable responses or cases where the trial was terminated due to other reasons. The larger green star on B cells compared to T cells for cladribine is intentional, as this drug has a predominant effect on B cells. Abbreviations: BAFF, B-cell Activating Factor; BAFFR, BAFF Receptor; BCR, B-cell Receptor; CXCL13, C-X-C Motif Chemokine Ligand 13; CXCR5, C-X-C Motif Chemokine Receptor 5; IL-2, Interleukin-2; IL-6, Interleukin-6; IL-6R, Interleukin-6 Receptor; IL-10, Interleukin-10; IL-17, Interleukin-17; IL-21, Interleukin-21; IL-22, Interleukin-22; IL-23, Interleukin-23; JAK, Janus Kinase; TCR, T-cell Receptor; TGF-β, Transforming Growth Factor Beta; TNF-α, Tumor Necrosis Factor Alpha; TNFR, Tumor Necrosis Factor Receptor.

As previously mentioned, although MG is the archetype for B cell-mediated autoimmune disorders, the loss-of-tolerance process is initiated by T cell help; in particular, CD4^+^ cells seem to be the main drivers in the pathogenesis of MG. Focusing on cytokine-mediated T-cell activity could be a promising target of MG treatment. However, to date, evidence comes mostly from preclinical studies (Figure 1).

One of the most explored is the role of IL-17 in MG pathogenesis, especially in EOMG, where higher levels of circulating Th1/17 cells associated with a pathogenic pro-inflammatory molecular profile have been found and correlated with disease severity [95]. IFN and IL-17 lead to a further reduction of Tregs and hyperactivation of Th17 cells, which enhances antibody production. An increase in Th17 populations has been observed in association with thymoma, regardless of the presence of MG. In EAMG, the moderation of disease severity following the ablation of IL-17 expression has recently been proven [96]. Based on this, monoclonal antibodies such as Secukinumab and Ixekizumab (anti-IL-17A), or those targeting the IL-17 receptor, including Brodalumab, could be considered as potential therapies for MG.

Because Th17 maturation is driven by IL-23 activation, a potential therapeutic approach targeting IL-23 in EAMG proved to be efficacious in ameliorating MG clinical manifestations. The treatment also reduced IL-17-related inflammation and anti-AChR IgG2b antibody production, activated transduction pathways involved in muscle regeneration, and improved the signal transduction at the neuromuscular junction [97]. In this context, Risankizumab, Guselkumab, and Tildrakizumab are antibodies directed against the p40 protein subunit present in IL-23 and are currently being used to treat other autoimmune diseases.

Decreased expression levels of the IL-23 receptor were also detected after JAK2 inhibitor treatments in EAMG. JAK regulates Th17/Treg balance by inhibiting both signaling pathways, and its inhibition improved clinical symptoms, increased AChR aggregation in the postsynaptic membrane, and reduced the immune response in EAMG models (including reducing pro-inflammatory cytokines and postsynaptic membrane complement deposition) [98].

Blockade of JAK1 and JAK2 suppresses effector T cells and B cells while Treg cell function is maintained, making these drugs potential candidates for the treatment of refractory autoimmune MG. Tofacitinib is an inhibitor of the enzymes JAK1 and JAK3 approved for the treatment of several chronic autoimmune diseases, such as rheumatoid arthritis and ulcerative colitis. A prospective, single-center, single-arm, single-blind pilot study is currently investigating the efficacy of tofacitinib in patients with refractory MG (ClinicalTrials.gov, NCT04431895 [99]); Ruxolitinib, a selective JAK1 and JAK2 inhibitor, induced clinical remission in a MuSK-positive MG patient who had undergone treatment for myelofibrosis [100].

Anti-TNF agents are widely employed in the field of immunology. In MG, a prospective pilot clinical trial evaluating etanercept in corticosteroid-dependent patients demonstrated efficacy in individuals with low serum levels of interferon-gamma and IL-6. In contrast, patients with elevated levels of these cytokines experienced clinical worsening, a finding consistent with several case reports [101,102]. Notably, the long-term use of anti-TNF agents has been associated with an uncertain risk of developing other immune-mediated pathologies. Therefore, further studies are warranted to clarify their therapeutic role in MG.

Similarly, although IFN-α therapy appears to be effective in suppressing EAMG, there are reports of therapy-induced or relapsed MG symptoms [41,103,104]. High-titer autoantibodies—predominantly of the IgG isotype—targeting IFN-α2 and IFN-ω (and IL-12) are commonly detected at the time of diagnosis in patients with MG [105]. Previous studies have demonstrated that IFN-β specifically induces the expression of the α-subunit of the AChR in thymic epithelial cells. Additionally, IFN-β promotes the upregulation of the chemokines CXCL13 and CCL21 by thymic epithelial cells and lymphatic endothelial cells, respectively [41]. These chemokines play a critical role in the formation of GCs and are markedly overexpressed in the hyperplastic thymus of MG patients exhibiting follicular hyperplasia, and corticosteroid treatment markedly reduced thymic CXCL13 overexpression [106]. Therapeutic strategies targeting the CXCL13–CXCR5 signaling axis are therefore of significant interest. A novel human monoclonal antibody, MAb 5261, designed to inhibit CXCL13-mediated signaling has recently been developed for the treatment of autoimmune diseases and has shown promising results in some experimental models (rheumatoid arthritis and a Th17-mediated murine model of multiple sclerosis) [107]. These findings highlight the potential relevance of this approach for the treatment of MG.

IL-6 is known to be elevated in the serum of treatment-naïve MG patients, is associated with clinical severity, and is reduced after immunosuppressive treatment [108]. EAMG mouse models with either acquired or congenital IL-6 deficiency exhibit resistance to disease development. In these models, blockade of the IL-6 receptor (IL-6R) led to clinical improvement, characterized by reduced muscle weakness, decreased IgG deposition at neuromuscular junctions, lower serum levels of AChR autoantibodies, and reductions in follicular helper T cells, Th17 cells, and plasma cells within lymph nodes [109,110]. Satralizumab, a monoclonal antibody targeting IL-6R, has been developed for this purpose. However, preliminary findings from a phase III clinical trial (LUMINESCE) (ClinicalTrials.gov, NCT04963270) [111] assessing its efficacy and safety in gMG have not demonstrated the anticipated clinical benefit [112]. Tocilizumab, a recombinant humanized monoclonal antibody against IL-6R, binds both membrane-bound and soluble IL-6 receptors, thereby inhibiting IL-6-mediated pro-inflammatory signaling. Some case reports and case series have suggested the efficacy and safety of tocilizumab in managing myasthenic symptoms, particularly in patients with refractory and very-late-onset MG (VLOMG) [113,114]. A phase II clinical trial evaluating tocilizumab in patients with generalized AChR antibody-positive MG is currently underway (ClinicalTrials.gov, NCT05067348) [115].

Other important regulators of immune homeostasis include B-cell lymphoma/leukemia 10 (BCL10), which has been implicated in both the activation and suppressive functions of Tregs. In experimental EAMG, microRNA miR-155-5p has been shown to inhibit Treg activation and immunosuppressive capacity by targeting BCL10, suggesting that BCL10 may represent a therapeutic target for the treatment of MG [116].

### 3.2. B Cell-Targeted Therapies

B cell-targeted therapies may modulate or remove the source of autoantibodies in MG patients through two different main mechanisms: functionally inactivating B cells or plasma cells or blocking their survival/differentiation (Figure 1) [48]. Several therapeutic targets have been identified in these contexts.

Controversial evidence comes from monoclonal antibodies against different clusters of differentiation on B cells, also depending on the antibody disease subtypes. Among these, the B-cell surface markers CD19 and CD20 have attracted attention due to their predominantly B cell-specific expression, enabling selective targeting with immunotherapeutic agents. Notably, CD19 is expressed earlier and more broadly than CD20 along the B-cell differentiation pathway—from pre-B cells to mature B cells and plasma cells—and remains detectable on late-stage ASCs even after CD20 expression is lost. The effectiveness of monoclonal antibody therapies targeting CD20 varies among gMG subtypes. Of note, current models propose that antibody production relies mostly on long-lived plasma cells in AChR-MG, while MuSK antibodies are mainly produced by short-lived plasmablasts [117].

In support of this, Rituximab, which targets B cells and short-lived plasma cell precursors, but not long-lived plasma cells (typically CD19^+^, CD20^−^), demonstrates a strong efficacy in MuSK-MG [118,119] but controversial efficacy in treating AChR-MG, in which removal of the thymus, where long-lived plasma cells reside, has been demonstrated to be clinically beneficial [16]. However, it is possible that in the early stages of AChR-MG, autoantibody production still derives mostly from short-lived plasma cells. In this case, Rituximab could be more effective if administered at disease onset, as demonstrated in the RINOMAX trial [120,121,122,123].

A recent systematic review [124] evaluated the role of rituximab in myasthenia gravis, including the RINOMAX [120] and BEAT-MG [125] trials. The review found that the long-term effects of rituximab on symptom severity and functional ability remain uncertain. While its steroid-sparing effect appears limited, rituximab probably reduces the risk of relapse requiring rescue therapy within nine months. Evidence on serious adverse events is of very low certainty, and there are currently no clear data to define the best dosing regimen or to identify which patient subgroups might benefit the most. Further trials on rituximab and other B cell-depleting therapies in different MG populations are therefore needed.

Two phase III randomized placebo-controlled trials are ongoing. The first, REFINE (ClinicalTrials.gov, NCT05868837) [126], is taking place in Italy and involves adults with MG and an MG-ADL score ≥ 5; it is expected to finish in 2025. The second, PROBE, is being conducted in France in adults with ocular MG and is scheduled for completion in 2028 (ClinicalTrials.gov, NCT06342544) [127].

Inebilizumab is a humanized IgG1κ that binds to and depletes CD19, inducing effective antibody-dependent cellular cytotoxicity and depleting almost all pre-B cells, including plasma cell precursors, thereby interfering more proximally with autoantibody production compared with anti-CD20 therapies. A phase III, double blind, placebo-controlled trial (MINT trial) (ClinicalTrials.gov, NCT04524273) [128] evaluated inebilizumab in 238 patients with AChR+ and MuSK+ MG [129]. Participants were randomized 1:1 to receive inebilizumab 300 mg IV on days 1 and 15 (with an additional dose at week 26 for AChR+ patients) or placebo, alongside a standardized corticosteroid taper. At week 26, inebilizumab achieved significantly greater improvements in MG-ADL and QMG compared with placebo in the overall population.

In both the AChR+ and MuSK+ subgroups, significant improvements in MG-ADL were observed at week 26. However, while the AChR+ subgroup also showed significant improvements in QMG, the difference in QMG score for the MuSK+ subgroup did not reach statistical significance. This suggests that although inebilizumab may be effective across antibody-defined subgroups, the strength of evidence is currently more robust in AChR+ patients.

At 52 weeks, benefits were sustained in the AChR+ subgroup, with greater improvements in MG-ADL and QMG and higher responder rates compared with placebo. The safety profile was favorable, with adverse events comparable to placebo and no new safety signals identified.

Fully humanized second-generation anti-CD20 agents—such as ocrelizumab, ofatumumab, obinutuzumab, and veltuzumab—may offer better tolerability compared to rituximab. A recent study reported the efficacy of ofatumumab in patients with AChR-MG, demonstrating improvement in clinical scales, along with a reduction in Tfh and Th17 cells, and decreased levels of IL-6, IL-21, and IL-17, in addition to B-cell depletion. However, current evidence remains limited, and no clinical trials in MG involving these agents are presently underway [130].

Among the various therapeutic targets under investigation in gMG, CD38 has emerged as a potential immunological target. CD38 is a cell surface glycoprotein highly expressed on both short- and long-lived plasma cells, plasmablasts, myeloid cells, and natural killer (NK) cells. Its expression is also induced on activated T cells and immunosuppressive cell populations, including regulatory T cells (Tregs). Mezagitamab (TAK-079), a fully humanized, subcutaneously administered anti-CD38 monoclonal antibody, which has received orphan drug designation for refractory multiple myeloma, was evaluated in a phase II, randomized, double-blind, placebo-controlled trial in patients with AChR- or MuSK-positive MG (ClinicalTrials.gov, NCT04159805) [131]. No patient discontinued the drug due to adverse events, and there were no statistically significant differences in terms of serious adverse events. However, the drug efficacy in terms of clinical scales has not already been investigated within a phase III clinical trial.

Iscalimab is a fully human, IgG1 Fc-silent, anti-CD40 monoclonal antibody that inhibits B-cell activation and immunoglobulin class-switching, germinal center formation, and the differentiation of B cells into plasma cells and plasmablasts, without depleting the B-cell population, with a good safety profile. CD40 is a transmembrane protein that is expressed as a receptor located on the membrane of APCs and follicular dendritic cells that specifically interacts with CD154 (also known as CD40 Ligand or CD40L), promoting the interaction with T helper lymphocytes. Moreover, a phase II, multicenter, double-blind, placebo-controlled trial (ClinicalTrials.gov, NCT02565576) [131] assessing the safety, tolerability, and efficacy of Iscalimab in moderate–severe gMG, even though it showed efficacy in MG-ADL improvement compared to placebo in non-thymectomized patients, did not meet the primary endpoint [132,133].

Noteworthy, T cells as well as dendritic cells produce exosomes, which are small granular substances secreted into the extracellular space that have a role in autoimmunity regulation. In recent years, dendritic cell-derived exosomes have received great attention in the treatment of autoimmune diseases and have undergone experimentation in EAMG. The use of statin-modified dendritic cell-derived exosomes (statin-Dex) resulted in the upregulation of CD40 and an increased expression of the AIRE gene, as well as the number of Treg cells in the thymus. On this basis, it could be considered as a future strategy for MG treatment [134,135].

Bortezomib, a proteasome inhibitor, was proven to be effective in vitro and partially in vivo. Its mechanism of action leads to apoptosis in cells that have a high protein turnover, such as plasma cells, which are sensitive to proteasome inhibition due to their constant secretion of antibodies. In the EAMG model, bortezomib reduced anti-AChR-antibody levels, prevented motor endplate damage, and induced clinical improvement. It has also shown promising results in the treatment of MuSK-MG, but results from randomized, double-blind clinical trials are still lacking [136]. A phase II study to investigate the effect of Bortezomib in patients with refractory MG, SLE, and rheumatoid arthritis was early terminated due to recruitment difficulties (ClinicalTrials.gov, NCT02102594) [137,138]. A selective inhibitor of the immunoproteasome (ONX-0914) has also proved to be efficacious in the classical EAMG model, ameliorating the severity of disease. ONX-0914 reduced autoantibody affinity and decreased Tfh cells and APCs; also, it reduced the percentage of Th17 cells and inhibited the secretion of IL-17 [139].

B lymphocyte stimulator (BlyS, also known as BAFF) and APRIL are instrumental in B-cell maturation and proliferation. BAFF, produced by monocytic and dendritic cells, is targeted by Belimumab, a monoclonal antibody that can bind receptors on different mature B cells: it was evaluated in a phase II trial in AChR-positive MG (ClinicalTrials.gov, NCT01480596) [140] but did not show any significant difference in clinical scores [141]. Telitacicept, similar to atacicept, is a transmembrane activator and calcium-modulator and cyclophilin ligand interactor (TACI)-based fusion protein that neutralizes both BAFF and APRIL, thereby suppressing B-cell survival and plasma cell differentiation. Early clinical evidence from a small retrospective study and case series suggested marked efficacy of telitacicept in improving symptoms of refractory MG patients [142,143].

These findings could be explained by the fact that in refractory MG, the proportion of regulatory B cells and Treg cells is reduced, and the expression of BAFF-R seems to be greater than in non-refractory MG (including thymoma-associated MG and seronegative MG patients) [144].

Building on these data, a phase II open-label study evaluated weekly subcutaneous telitacicept (160 or 240 mg) as add-on to standard therapy and demonstrated safety, good tolerability, and reduced clinical severity throughout the study period [145].

More recently, a randomized, double-blind, placebo-controlled phase III trial in China (ClinicalTrials.gov, NCT05737160) [146] confirmed these results, showing robust clinical efficacy: at week 24, telitacicept achieved greater improvements in MG-ADL and QMG compared with placebo, with a similar incidence of adverse events and fewer infection-related events. These findings highlight telitacicept as a promising novel therapy for gMG, especially in refractory patients. A global phase III multicenter trial (ClinicalTrials.gov, NCT06456580) [147] is currently underway to further validate the efficacy and safety in broader international populations.

Tolebrutinib, an oral Bruton’s tyrosine kinase inhibitor (BTKi) mostly used for B-cell malignancies, was also being investigated for gMG in a phase III clinical trial (URSA trial) (ClinicalTrials.gov, NCT05132569) [148]. BTK phosphorylation leads to increased B-cell proliferation, differentiation, and survival and is a critical element in B-cell signaling. The trial was interrupted due to cases of drug-induced liver injury—particularly in patients with preexisting liver risk—and a strategic reassessment by the pharmaceutical company [149]. At present, tolebrutinib is no longer being developed for MG, but research continues in other conditions such as multiple sclerosis, where it has shown promising results in slowing disease progression [150]. A phase III trial investigating the efficacy, safety and tolerability of Remibrutinib is ongoing (ClinicalTrials.gov, NCT06744920). 

Cladribine (2-chlorodeoxyadenosine, 2-CdA) is a synthetic purine nucleoside analog that acts as a prodrug and is intracellularly phosphorylated by deoxycytidine kinase into its active triphosphate form. Once incorporated into DNA, it interferes with DNA synthesis and repair, leading to apoptosis. Because lymphocytes have particularly high deoxycytidine kinase activity and low 5′-nucleotidase activity, they are especially sensitive to cladribine, resulting in preferential depletion of B and T cells, with a particular predilection for memory B cells [151].

The mechanism of cladribine is of particular interest in antibody-mediated diseases such as MG because it transiently but profoundly reduces autoreactive B- and T-cell populations, followed by gradual immune reconstitution. This “immune reset” model has already been validated in multiple sclerosis, where short treatment cycles of oral cladribine provide durable disease control with limited cumulative toxicity [152,153].

The only published clinical evidence for cladribine in MG is a small, open-label pilot study involving 13 patients with refractory disease, which reported clinical improvement and steroid-sparing effects over four months, without major adverse events during the study period. However, this study was limited by its small sample size, open-label design, and lack of a control group [154]. A phase III study is now ongoing [155] (ClinicalTrials.gov, NCT06463587) [156].

### 3.3. Other B and T Cell Depletion Therapies

Chimeric antigen receptor T-cell (CAR-T) therapy, originally developed for hematologic malignancies, is now being investigated in autoimmune diseases, thanks to technological advances aimed at improving precision and safety. CAR-T involves isolating a patient’s T cells, genetically modifying them to express a synthetic receptor targeting a specific antigen, expanding them ex vivo, and reinfusing them into the patient [157]. In autoimmune settings, CAR-T cells are typically designed to eliminate B cells or plasma cells that produce pathogenic autoantibodies by targeting surface molecules such as CD19 or BCMA [158].

However, the conventional approach raises concerns due to prolonged immunosuppression and the risk of severe immune-mediated adverse events, particularly cytokine release syndrome (CRS). To address these limitations, next-generation platforms such as chimeric autoantibody receptor T-cell (CAAR-T) and RNA-based CAR-T (rCAR-T) have been developed.

CAAR-T therapy is an emerging targeted immunotherapy that involves engineering T cells to express chimeric autoantibody receptors capable of selectively targeting autoreactive B cells—specifically those that produce harmful autoantibodies. Unlike traditional CAR-T therapies that aim to eliminate the entire B-cell lineage, CAAR-T cells are designed to remove only the autoreactive B-cell clones involved in disease development. This specificity is achieved by displaying the relevant autoantigen on the surface of the CAAR-T cells, enabling recognition and engagement by the B-cell receptors (BCRs) of the pathogenic clones.

MG represents a paradigmatic model of antibody-mediated autoimmunity, making it particularly suitable for testing CAR-T and CAAR-T approaches. In this context, MuSK-positive MG is considered an ideal candidate for CAAR-T development because MuSK autoantibodies are IgG4, target a single well-defined epitope, and are pathogenic by blocking receptor clustering. By contrast, AChR-positive MG poses greater challenges for CAAR-T engineering, as autoantibodies recognize multiple epitopes across the pentameric AChR complex, complicating the design of a single effective CAAR construct [159].

A phase I, open-label, safety and dose-escalation clinical trial is currently underway to evaluate MuSK-CAART in patients with anti-MuSK antibody-positive MG (ClinicalTrials.gov, NCT05451212) [160]. Preclinical studies in an EAMG model have demonstrated that MuSK-CAART therapy can selectively deplete anti-MuSK B cells, leading to a reduction in anti-MuSK IgG levels without altering total B-cell counts or overall IgG concentrations, indicating a targeted immunomodulatory effect [161].

Meanwhile, rCAR-T platforms, such as Descartes-08, use mRNA transfection to induce transient CAR expression, allowing repeated dosing, improved pharmacokinetics, and a reduced risk of long-term toxicity. Descartes-08 targets BCMA and has shown excellent safety and sustained clinical benefit in a phase I/II trial in severe MG [162], with no CRS, neurotoxicity, or dose-limiting toxicities, leading to an ongoing phase IIb trial (ClinicalTrials.gov, NCT04146051) [163]. In this early trial, many patients achieved minimal manifestation status with durable responses lasting over 9 months after the last infusion, further supporting the potential of rCAR-T as a safer and more flexible cellular platform.

Additionally, a case report has demonstrated the safety and efficacy of a CD19-targeting CAR-T therapy in a patient with severe, treatment-refractory MG, including resistance to rituximab [164].

Furthermore, two phase I/II clinical trials are planned to assess the safety and efficacy of CD19-targeting CAR-T cell candidates (KYV-101 and CABA-201) for B cell-driven autoimmune disorders (ClinicalTrials.gov, NCT06193889 [165] and NCT06359041 [166]), aiming to provide effective and durable B-cell depletion.

A major concern surrounding the use of CAR-T and CAAR-T cell therapies is the risk of life-threatening adverse events, as observed in patients treated for hematologic malignancies. Chief among these is cytokine release syndrome (CRS), which has emerged as the most severe complication. CRS symptoms can range from mild, such as fever and anorexia, to more serious manifestations, including hypotension and hypoxemia, often resulting from capillary leak syndrome [167]. The pathogenesis of CRS is largely driven by IL-6 signaling, and therapeutic agents such as tocilizumab (an IL-6 receptor antagonist) and corticosteroids are effective in mitigating these effects. Importantly, no CRS or neurotoxicity was observed with Descartes-08 in MG, underscoring the improved safety of RNA-based approaches compared with conventional CAR-T.

Given the limitations of CAR-T cell therapies—including preconditioning requirements, high costs, and manufacturing delays—bispecific antibodies have emerged as a promising off-the-shelf alternative. Among these, blinatumomab, a CD19xCD3 bispecific T-cell engager originally developed for acute lymphoblastic leukemia, redirects cytotoxic T cells to eliminate autoreactive B cells by simultaneously binding CD3 on T cells and CD19 on B cells. This dual binding promotes targeted B-cell apoptosis via perforin- and granzyme-mediated mechanisms. Notably, blinatumomab achieves deeper and more sustained B-cell depletion compared to rituximab, and a recent report of two cases has identified it as a therapeutic option in treatment-refractory MG, although further investigations in clinical trials are needed [168].

It is worth noting that hematopoietic stem cell transplantation (HSCT) remains the only therapy currently capable of eradicating all autoreactive T and B cells, thereby enabling a full immunologic reset. As such, HSCT represents the only intervention with the potential to induce long-term, treatment-free remission, effectively approaching a functional cure. Despite the risks associated with this approach, including systemic toxicity, infertility, and a heightened susceptibility to infections, HSCT is being increasingly considered for patients with severe and treatment-refractory autoimmune diseases, including MG. All reported cases of HSCT in MG have resulted in complete and sustained remission [169].

### 3.4. Gene Therapies

Gene therapies targeting microRNAs have been investigated primarily in EAMG models, while no human clinical trials have yet tested miR-based approaches in MG.

In EAMG mice, targeted reduction of miR-125a-5p using gene silencing approaches led to improved muscle strength, increased Foxp3 expression, the expansion of Tregs, and elevated levels of Treg-associated cytokines (IL-10, TGF-β1), while suppressing pathogenic T-cell proliferation. Conversely, overexpression of miR-125a-5p worsened disease features, indicating that miR-125a-5p negatively regulates Treg function by targeting Foxp3 and contributes to disease pathogenesis [170].

Similarly, in EAMG rats, lentiviral-mediated overexpression of miR-145 in CD4^+^ T cells reduced disease severity, decreased Th17 cell responses, and lowered anti-acetylcholine receptor antibody levels. Mechanistically, miR-145 directly suppressed CD28 and NFATc1 expression, leading to reduced T-cell activation and pathogenicity [171].

Silencing of a regulatory nucleic acid molecule, miR-146, has shown promise in alleviating MG symptoms [172].

Notably, miRNAs have shown specific expression patterns in different patient serum subgroups, especially identifying distinct profiles in generalized AChR+ EOMG, LOMG, MuSK-MG, and ocular MG, where higher levels of certain miRNAs can distinguish patients who will later develop generalization from those who remain ocular [173]. However, these remain biomarkers under investigation and not therapeutic targets in clinical practice.

Monarsen (EN101) is an antisense oligonucleotide that targets acetylcholinesterase (AChE) mRNA, specifically the “readthrough” isoform (AChE-R), and is under investigation as a symptomatic treatment for MG. Unlike conventional acetylcholinesterase inhibitors, Monarsen selectively reduces AChE-R expression, which is upregulated in MG and experimental autoimmune myasthenia gravis (EAMG), without significantly affecting the synaptic AChE-S isoform. This targeted approach aims to enhance neuromuscular transmission and reduce muscle fatigue while minimizing cholinergic side effects commonly seen with non-selective AChE inhibitors [174].

In both EAMG models and early-phase clinical studies, Monarsen has demonstrated improvement in muscle strength and neuromuscular function, with a favorable safety profile and the absence of significant cholinergic toxicity [175].

Small interfering RNAs (siRNAs) have demonstrated therapeutic potential in EAMG models but are not yet in clinical use for MG. In EAMG, siRNA-mediated knockdown of complement component C2 significantly improved muscle strength, preserved functional AChRs, and reduced complement deposition at the neuromuscular junction, supporting the feasibility of targeting complement pathways with siRNA to ameliorate disease severity [176]. This was also true for investigational siRNA targeting C5 [177]. More recently, B cell-specific monoclonal antibody–siRNA conjugates targeting BAFF-R (CD268) and BCMA (CD269) on B-cell subsets reduced pathogenic AChR autoantibody levels and improved clinical outcomes in EAMG [178]. While these preclinical findings are encouraging, translation into human trials remains to be established.

### 3.5. Anti-FcRn Drugs

The neonatal Fc receptor (FcRn) is a molecule that is structurally similar to MHC class I and is expressed across various cell types. Its significance as a target for immunomodulatory therapies stems from its critical role in the recycling of immunoglobulin G (IgG). FcRn facilitates the transcytosis of IgG (and albumin) across endothelial cells, thereby markedly extending their half-life. However, a consequence of this mechanism is the prolonged persistence of pathogenic autoantibodies in IgG-mediated autoimmune disorders. Therapeutic agents targeting FcRn promote the accelerated degradation of endogenous IgG, including disease-associated autoantibodies [179,180].

Efgartigimod and rozanolixizumab are currently the only FcRn inhibitors approved for the treatment of gMG, with regulatory endorsement supported by robust phase III clinical trial data. These trials demonstrated significant improvements in both the MG-ADL and QMG scores compared to placebo, with efficacy observed in both AChR and MuSK antibody-positive subgroups [10,24].

Batoclimab and nipocalimab are investigational FcRn antagonists that have successfully completed the pivotal efficacy phase of their respective phase III trials (ClinicalTrials.gov, NCT05403541, and NCT04951622) [181,182]^,^ demonstrating significant clinical benefit on validated disease activity scales. Both studies remain formally ongoing to allow long-term extension and safety follow-up [183,184].

Meta-analyses suggest that FcRn inhibitors as a drug class are associated with meaningful enhancements in functional outcomes and health-related quality of life, and they generally exhibit a favorable safety profile. The most frequently reported adverse events include headache and infections, while some agents are associated with transient hypoalbuminemia and hypercholesterolemia [185,186].

Emerging real-world evidence indicates variability in treatment response among different MG subtypes. Patients with TAMG appear more likely to discontinue anti-FcRn therapies [27]. Conversely, individuals with higher baseline disease activity and worse clinical scores tend to derive more substantial therapeutic benefit. Notably, patients with anti-MuSK antibodies or seronegativity may experience a more pronounced response compared to those with anti-AChR antibodies, potentially due to the functionally monovalent nature of the former’s pathogenic mechanism [187].

### 3.6. Complement Inhibitors

Complement inhibitors are targeted immunotherapies that block activation of the complement cascade, thereby preventing tissue damage at the neuromuscular junction. Inhibitors such as eculizumab, ravulizumab, and zilucoplan binding to complement component C5 have been approved. These agents have shown significant clinical benefit in reducing disease severity and improving quality of life in patients with anti-AChR gMG [9,23,25]. The binding of eculizumab and ravulizumab depends on the presence of an arginine residue at position 885 of the complement protein C5. A mutation substituting this arginine with histidine (p.Arg885His) can confer resistance to eculizumab and ravulizumab. In contrast, zilucoplan is not affected by this polymorphism [188].

**Figure 2:**
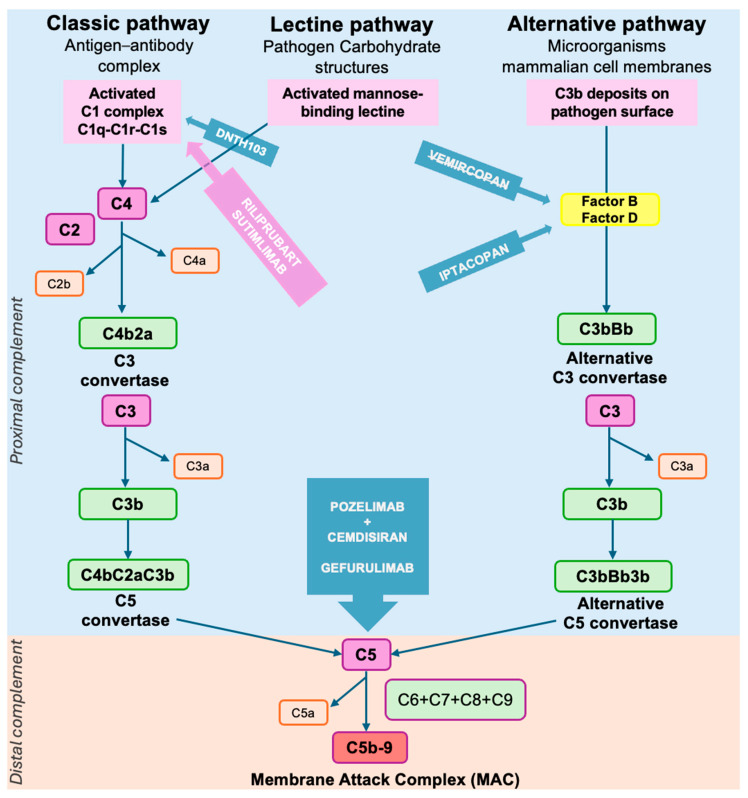
Complement cascade-targeted therapies. This figure shows the key targets and corresponding therapies in the complement cascade. The figure is intentionally schematic to facilitate understanding and highlight the main therapeutic strategies. Agents already used in other diseases with targets potentially relevant for MG are highlighted in pink; drugs evaluated in RCTs are highlighted in blue. The white line crossing the drug name indicates agents that did not show favorable responses.

Several new therapeutic targets and agents are currently under development, aiming to overcome resistance to standard approved complement inhibitors and to identify alternative, effective modes of administration (Figure 2).

No approved therapies targeting C1q, C1s, or C1r currently exist for MG, although some are approved for other indications. Available evidence is primarily derived from preclinical studies. In EAMG models, treatment with an anti-C1q antibody significantly reduced disease severity and improved grip strength. This benefit was associated with reduced formation of C3 and prevention of MAC deposition. Additionally, anti-C1q therapy moderately decreased IL-6 production and lymph node cellularity, suggesting a broader immunomodulatory role for C1q in cellular immune responses [189].

Sutimlimab and Riliprubart are humanized monoclonal antibodies that target active C1s in the classical complement pathway. While neither has been investigated in MG-specific trials, sutimlimab is approved for the treatment of cold agglutinin disease [190], and riliprubart has been evaluated in a phase Ib study for cold agglutinin disease [191] and in a phase III trial (ClinicalTrials.gov, NCT06290128) [192] for patients with chronic inflammatory demyelinating polyneuropathy (CIDP). Their established or ongoing clinical use in complement-mediated disorders provides a mechanistic rationale for potential repurposing in MG, although this remains to be formally tested. A phase II trial to investigate safety and efficacy of DNTH103, an active C1s inhibitor, is ongoing (ClinicalTrials.gov, NCT06282159) [193].

The phase III trial in MG of the oral Factor D inhibitor, vemircopan [with the same mechanism of action as danicopan [194], a molecule already approved as an add-on therapy for Paroxysmal Nocturnal Hemoglobinuria (PNH)], has been terminated early by the sponsor due to lack of efficacy based on primary and secondary endpoint results against placebo (ClinicalTrials.gov, NCT05218096) [195].

Iptacopan is an oral complement Factor B inhibitor recently approved by the FDA for the treatment of PNH, and it appears to be effective even in patients who are refractory to eculizumab [196]. It is currently being investigated in a phase III clinical trial (ClinicalTrials.gov, NCT06517758) [197] as a promising oral therapy for patients with anti-AChR-positive myasthenia gravis.

Cemdisiran, an N-acetylgalactosamine-conjugated small interfering RNA that suppresses liver production of C5, and pozelimab, a fully human monoclonal antibody inhibitor of C5, are now under evaluation in a phase III study (ClinicalTrials.gov, NCT05070858) [198]. They both have a subcutaneous administration, as well as Gefurulimab, a monoclonal antibody (ClinicalTrials.gov, NCT05556096) [199].

**Table 1:** Summary of biological targets and therapeutic agents in MG.

Target Group	Drug	Mechanism/ Target	Subtype of MG	Clinical Trial Status/ Approval	Key Highlights
T cell- and cytokine-targeted therapies	**Tofacitinib**	JAK1/3	AChR+ MG	NCT04431895 [99] *ongoing*	Pilot study: no data available.
	**Ruxolitinib**	JAK1/2	MuSK+ MG	-	Case report: MuSK-MG clinical remission [100].
	**Etanercept**	TNF-α	AChR+ MG	-	Pilot study: mixed results depending on cytokine profile [101].
	**Satralizumab**	IL-6R	AChR+ MG MuSK+ MG LRP4+ MG	NCT04963270 [111] *terminated*	Phase III trial: Satralizumab was well tolerated and resulted in small improvements in patient-reported and clinician-reported outcomes compared with placebo at week 24 in AChR+ MG patients [112].
	**Tocilizumab**	IL-6R	AChR+ MG	NCT05067348 [115] *ongoing*	Case series: benefit in refractory/VLOMG [113,114]. Phase II trial: ongoing.
	**miR-155-5p**	BCL10		-	Preclinical evidence: inhibits Treg activation, immunosuppressive capacity [116].
B cell-targeted therapies	**Rituximab**	CD20	AChR+ MG MuSK+ MG	NCT05868837 [126] *ongoing* NCT06342544 [127] *ongoing*	Highly effective in MuSK+ [117,118]. Mixed results in AChR+ [118,119,120,121,122,123,124]: Rituximab could be more effective if administered at disease onset [119]. Currently, two phase III trials are ongoing [126,127].
	**Inebilizumab**	CD19	AChR+ MG MuSK+ MG	NCT04524273 [128] *ongoing*	Phase III trial: effective in both AChR+ and MuSK+, stronger data in AChR+ cohort [129].
	**Ofatumumab**	CD20	AChR+ MG	-	Pilot study: improvement in clinical scales at 1 and 3 months [130].
	**Mezagitamab (TAK-079)**	CD38	AChR+ MG MuSK+ MG	NCT04159805 [131] *completed*	Phase II trial: negative results posted [131].
	**Iscalimab**	CD40	AChR+ MG MuSK+ MG	NCT02565576 [131] *completed*	Phase II trial: primary endpoint not met [132].
	**Belimumab**	BAFF	AChR+ MG MuSK+ MG	NCT01480596 [140] *completed*	Phase II trial: any significant difference in clinical scores [141].
	**Telitacicept**	BAFF/APRIL	AChR+ MG MuSK+ MG	NCT06456580 [147] *ongoing*	Phase II and III in China: positive results [145,146]. Global phase III: ongoing [147].
	**Tolebrutinib**	BTK	AChR+ MG MuSK+ MG Seronegative MG	NCT05132569 [148] *terminated*	Phase III trial: terminated early by the sponsor for strategic reasons [149].
	**Remibrutinib**	BTK	AChR+ MG MuSK+ MG Seronegative MG	NCT06744920 [155]	Phase III trial: ongoing
Plasma cell-directed therapies	**Bortezomib**	Proteasome	AChR+ MG	NCT02102594 [138] *terminated*	Phase II trial: terminated early due to recruitment difficulties [137,138].
	**ONX-0914**	Immunoproteasome		-	Preclinical evidence: reduced autoantibody affinity and the percentage of Th17 cells; inhibited the secretion of IL-17 [139].
Other depletion/immune reset	**Cladribine**	Purine analog, lymphocyte depletion and immune reset	AChR+ MG MuSK+ MG LRP4+ MG	NCT06463587 [156] *ongoing*	Pilot study: clinical improvement [154]. Phase III trial: ongoing [156].
	**HSCT**	Immune ablation/reconstitution	AChR+ MG	-	Case series: long-term remission; high toxicity [169].
Cellular therapies	**CAR-T**	CD19	AChR+ MG	NCT06193889 [165] *ongoing* NCT06359041 [166] *ongoing*	Case report in MG [164]. Phase II and Phase I/II: ongoing.
	**CAAR-T** (MuSK)	MuSK auto-reactive B cells	MuSK+ MG	NCT05451212 [160] *ongoing*	Preclinical evidence, phase I ongoing [160,161].
	**rCAR-T** (Descartes-08)	BCMA	AChR+ MG MuSK+ MG Seronegative MG	NCT04146051 [163] *ongoing*	Phase I/II trial: safe and sustained clinical benefit [162]. Phase IIb: ongoing [163].
Bispecific antibodies	**Blinatumomab (CD19 × CD3)**	T cell engager	AChR+ MG	-	Two cases of refractory MG: good response [168].
FcRn antagonists	**Efgartigimod**	FcRn	AChR+ MG MuSK+ MG Seronegative MG	Approved	In-label for AChR+ MG [10].
	**Rozanolixizumab**	FcRn	AChR+ MG MuSK+ MG	Approved	In-label for AChR+ MG and MuSK+ MG [24].
	**Batoclimab**	FcRn	AChR+ MG MuSK+ MG	NCT05403541 [181] *ongoing*	Phase III trial: significant clinical improvement [183].
	**Nipocalimab**	FcRn	AChR+ MG MuSK+ MG	NCT04951622 [182] *ongoing*	Phase III trial: significant clinical improvement [184].
Complement inhibitors	**Eculizumab**	C5	AChR+ MG	Approved	In-label for AChR+ gMG [9].
	**Ravulizumab**	C5	AChR+ MG	Approved	In-label for AChR+ gMG [23].
	**Zilucoplan**	C5	AChR+ MG	Approved	In-label for AChR+ gMG [25].
	**DNTH103**	C1s	AChR+ MG	NCT06282159 [193] *ongoing*	Phase II trial: ongoing.
	**Iptacopan**	Factor B	AChR+ MG	NCT06517758 [197] *ongoing*	Phase III trial: ongoing.
	**Vemircopan**	Factor D	AChR+ MG	NCT05218096 [195] *terminated*	Phase III trial: terminated early by the sponsor.
	**Cemdisiran + Pozelimab**	C5	AChR+ MG	NCT05070858 [198] *ongoing*	Phase III trial: ongoing.
	**Gefurulimab**	C5	AChR+ MG	NCT05556096 [199] *ongoing*	Phase III trial: ongoing.

## 4. Conclusions

In the past few years, important milestones in MG management have emerged. Several molecules targeting specific molecular mechanisms are changing the lives of people with MG, allowing for effective control of disease burden, especially in those with gMG resistant to traditional therapies. The excitement from these results has sparked the development of different research paths, with some focusing on relatively new molecular pathways in MG. As one of the best examples of antibody-mediated diseases, MG might seem simple to treat and manage. However, beneath the surface, a complex array of molecular and cellular mechanisms defines each major phenotype, influencing treatment decisions. Additionally, the variability in responses to new biological treatments shows that differences between individuals exist even within the same MG subtype. Factors such as disease duration, age, previous treatments, and polymorphisms in molecular targets are becoming recognized as important, but they provide limited insight into the overall complexity. Likely, the failure of some clinical trials targeting promising molecular markers is due to choosing the wrong MG subpopulation. More research into the molecular signatures of MG phenotypes is crucial to better guide the design of future clinical trials.

## Data Availability

Not applicable.
